# Obeticholic Acid Reduces Kidney Matrix Metalloproteinase Activation Following Partial Hepatic Ischemia/Reperfusion Injury in Rats

**DOI:** 10.3390/ph15050524

**Published:** 2022-04-24

**Authors:** Giuseppina Palladini, Marta Cagna, Laura Giuseppina Di Pasqua, Luciano Adorini, Anna Cleta Croce, Stefano Perlini, Andrea Ferrigno, Clarissa Berardo, Mariapia Vairetti

**Affiliations:** 1 Department of Internal Medicine and Therapeutics, University of Pavia, 27100 Pavia, Italy; giuseppina.palladini@unipv.it (G.P.); marta.cagna02@universitadipavia.it (M.C.); lauragiuseppin.dipasqua01@universitadipavia.it (L.G.D.P.); stefano.perlini@unipv.it (S.P.); andrea.ferrigno@unipv.it (A.F.); 2 Internal Medicine Fondazione IRCCS Policlinico San Matteo, 27100 Pavia, Italy; 3 Intercept Pharmaceuticals, San Diego, CA 92121, USA; LAdorini@interceptpharma.com; 4 Institute of Molecular Genetics, Italian National Research Council (CNR), 27100 Pavia, Italy; annacleta.croce@igm.cnr.it; 5 Emergency Department Fondazione IRCCS Policlinico San Matteo, 27100 Pavia, Italy

**Keywords:** kidney, obeticholic acid, ischemia/reperfusion, liver, metalloproteinases

## Abstract

We have previously demonstrated that the farnesoid X receptor (FXR) agonist obeticholic acid (OCA) protects the liver via downregulation of hepatic matrix metalloproteinases (MMPs) after ischemia/reperfusion (I/R), which can lead to multiorgan dysfunction. The present study investigated the capacity of OCA to modulate MMPs in distant organs such as the kidney. Male Wistar rats were dosed orally with 10 mg/kg/day of OCA (5 days) and were subjected to 60-min partial hepatic ischemia. After 120-min reperfusion, kidney biopsies (cortex and medulla) and blood samples were collected. Serum creatinine, kidney MMP-2, and MMP-9-dimer, tissue inhibitors of MMPs (TIMP-1, TIMP-2), RECK, TNF-alpha, and IL-6 were monitored. MMP-9-dimer activity in the kidney cortex and medulla increased after hepatic I/R and a reduction was detected in OCA-treated I/R rats. Although not significantly, MMP-2 activity decreased in the cortex of OCA-treated I/R rats. TIMPs and RECK levels showed no significant differences among all groups considered. Serum creatinine increased after I/R and a reduction was detected in OCA-treated I/R rats. The same trend occurred for tissue TNF-alpha and IL-6. Although the underlying mechanisms need further investigation, this is the first study showing, in the kidney, beneficial effects of OCA by reducing TNF-alpha-mediated expression of MMPs after liver I/R.

## 1. Introduction

Obeticholic acid (OCA, INT-747) is a bile acid-derived FXR agonist currently in phase III trials for the treatment of NASH that has already shown its potential for treating hepatic steatosis, inflammation, and fibrosis while increasing insulin sensitivity [1]. Recently, the REGENERATE study showed that the administration of OCA in NASH patients not only ameliorates liver injury and fibrosis but also improves several health-related quality of life (HRQoL) domains [2].

Fibrosis is a type of chronic organ failure, resulting in the excessive secretion of extracellular matrix (ECM). Matrix metalloproteinases (MMPs), the main group of ECM-degrading enzymes, are considered to be a potential target for fibrosis treatment [3].

Tissue Inhibitor of Metalloproteinases (TIMP-1), the major endogenous regulator of MMP-9, plays a protective function in the control of survival and proliferation of liver cells during I/R injury [4]. MMP-2 and MMP-9 are reduced by interaction with the reversion-inducing-cysteine-rich protein with kazal motifs (RECK), a transformation-suppressor gene that regulates the expression of several MMPs and is involved in the inhibition of the tumor invasion and metastasis process [5].

We have recently shown that OCA decreases MMP-2 and MMP-9 activity in tissue and bile obtained from livers submitted to partial ischemia/reperfusion (I/R) injury [6]. This is associated with the ability of OCA to restore inhibitors of MMP-2 and MMP-9 such as RECK and TIMPs (TIMP-1 and TIMP-2) decreased by ischemic insult [6].

The I/R injury in one organ can also lead to multiorgan dysfunction, that is tissue damage in remote organs away from the body district where the I/R damage is taking place. For example, I/R intestine damage has been described to cause multiple organ dysfunction due to uncontrolled production and release of cytokines and other proinflammatory molecules [7]. Hepatic I/R injury may also cause damage to remote organs such as the kidney, heart, and lung. In addition to liver dysfunction as a consequence of hepatic reperfusion, many remote organs seem to be influenced during this process as well [8]. We have previously documented that moderate acute hepatic ischemia (30 min) followed by reperfusion (60 min) increases MMPs activity not only in the ischemic liver region but also in the lung, associated with histological damage in the liver, lung, and kidney [9]. Clinical studies have reported that, in patients with acute liver failure induced by I/R, the incidence of acute kidney injury (AKI) ranges from 40 to 85%, and up to 95% in liver transplantation [10]. Indeed, damaged liver tissue releases destructive proinflammatory cytokines and oxygen-derived radicals into the circulation that are likely causing further damage to remote organs [11]. Defined as multiple organ dysfunction syndrome (MODS) or multiple organ failure (MOF), this event is an important cause of death in surgical intensive care units. In particular, MODS includes altered organ function in sepsis, septic shock, and systemic inflammatory response syndrome [8].

Most of the current knowledge on liver I/R and remote organ injury derives from experimental studies as well as from the assessment of the efficacy of innovative therapeutic strategies. Based on the above reports, we designed a study in rats to investigate whether liver ischemia (60 min) followed by reperfusion (120 min) affect the function and the structure of remote organs such as kidney, via modulation of ECM remodeling. Using these models, we also tested if OCA treatment is able to counteract remote damage via restoration of MMPs and their specific inhibitors, TIMP-1, TIMP-2, and RECK.

## 2. Results

### 2.1. Hepatorenal Syndrome after Liver I/R

After a consensus conference in 1978, hepatorenal syndrome, renal insufficiency progressing in the presence of severe liver diseases, and the absence of recognized nephrotoxic agents were defined by plasma creatinine ≥1.5 mg/dL [12]. In the present study, the evaluation of serum creatinine showed a 1.8-fold increase confirming the induction of hepatorenal syndrome after liver I/R injury (Table 1). Recently, the International Club of Ascites (ICA) has adopted the concept of acute kidney injury (AKI) which was developed originally to be used in critically-ill patients. AKI is defined as an increase in creatinine of at least 0.3 mg/dL (26 μmol/L) and/or ≥50% from baseline, within 48 h [13].

Our results also demonstrate that rats subjected to liver I/R developed severe liver dysfunction after 1 h hepatic ischemia followed by 2 h reperfusion, with significantly higher serum levels of AST, ALT, ALP, total and direct bilirubin compared to sham-operated animals (Table 1).

Moreover, we demonstrate a direct relationship between the severity of liver dysfunction (ALT) and the degree of kidney damage (creatinine) after I/R (Table 1). Our data are in agreement with those reported by Lee HT (2009) who studied renal dysfunction in mice after liver I/R injury: animals subjected to 60 min ischemia showed a direct and linear relationship between plasma ALT and creatinine levels after liver I/R [14].

### 2.2. Changes in MMPs, TIMPs, and RECK in Kidney Cortex and Medulla after Liver I/R

**Figure 1:**
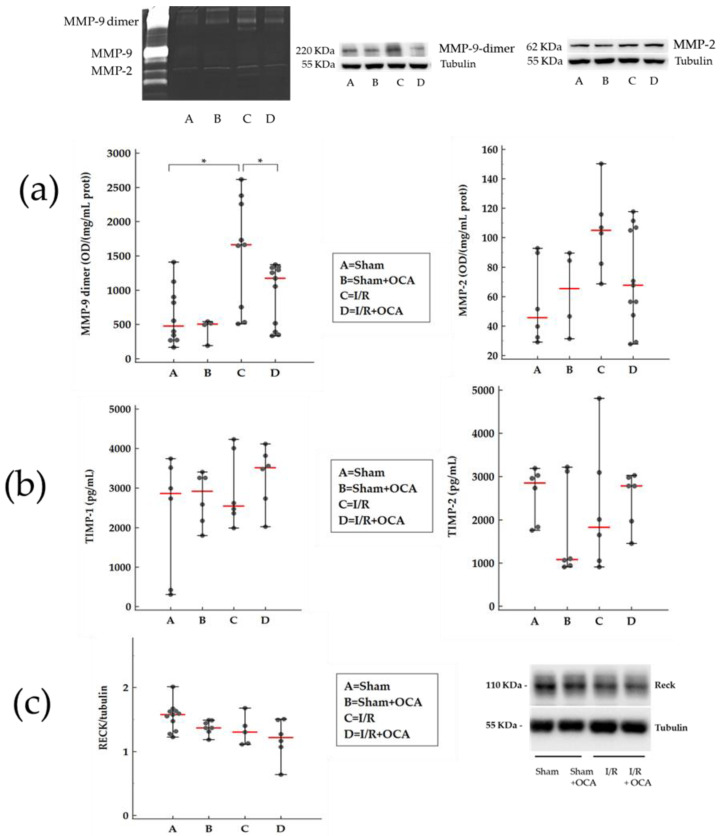
OCA treatment decreases MMP-9-dimer activity in kidney cortex after liver I/R. Animals were orally administered 10 mg/kg/day of OCA in methylcellulose 1% vehicle for 5 days (*n* = 12) or vehicle alone (*n* = 12). (**a**) Representative of MMP zymography and Western blot; gelatinolytic activities of MMP (MMP-9-dimer and MMP-2) quantified by densitometry. (**b**) TIMPs (TIMP-1 and TIMP-2) by Elisa kits and (**c**) RECK expression levels, by Western Blots, were determined in kidney cortex of rats submitted to partial liver ischemia (60 min) followed by reperfusion (120 min). Sham-operated animals were subjected to the same procedure without clamping the vessels. *n* = 6 rats/group. Results are expressed as median value (line red) ± error bar (1–99 percentiles) (black dot). One-way ANOVA with Tukey–Kramer test (TIMP-1 and RECK); Kruskal–Wallis test with Conover test (MMP-9-dimer, MMP-2, TIMP-2). * *p* < 0.05 versus I/R rats.

We have already reported that liver I/R injury is associated with MMP activation with profound effects on tissue integrity [15]; this event also influences the function of many remote organs [9,16]. In the present study, we evaluated the MMP-9-dimer activity in the kidney cortex after hepatic ischemia (60 min) followed by reperfusion (120 min): a significant increase in MMP-9-dimer activity was found in the I/R group compared with sham-operated rats (Figure 1a). A reduction in cortex MMP-9-dimer activity was detected in OCA-treated I/R rats compared with vehicle-treated I/R rats (Figure 1a).

**Figure 2:**
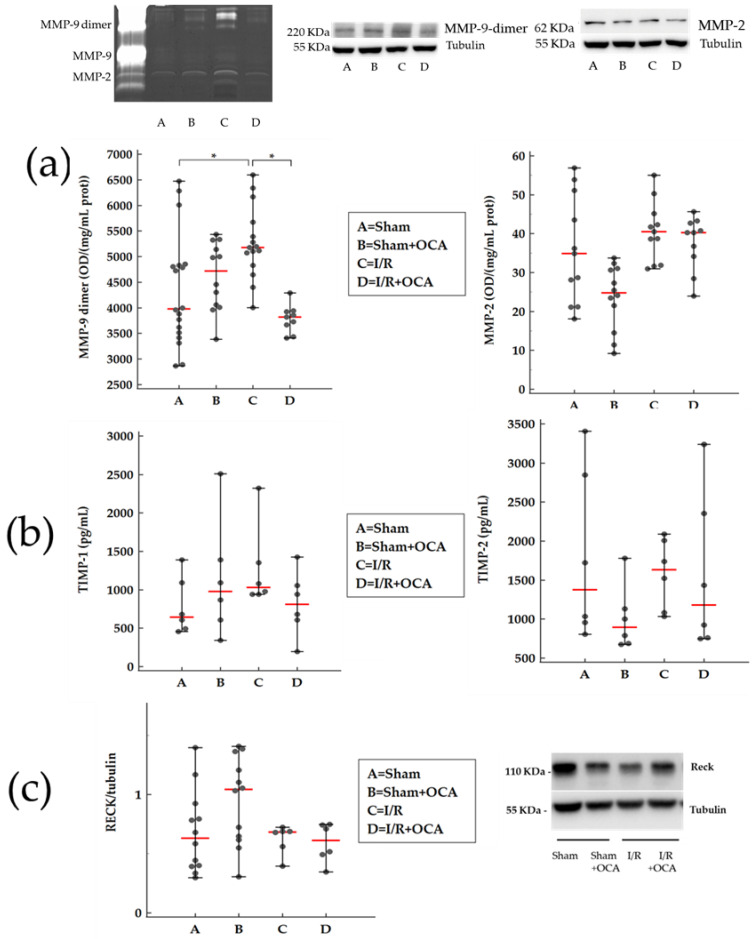
OCA treatment decreases MMP-9-dimer activity in kidney medulla after liver I/R. Animals were orally administered 10 mg/kg/day of OCA in methylcellulose 1% vehicle for 5 days (*n* = 12) or vehicle alone (*n* = 12). (**a**) Representative of MMP zymography and Western blot; gelatinolytic activities of MMP (MMP-9-dimer and MMP-2) quantified by densitometry. (**b**) TIMPs (TIMP-1 and TIMP-2) by Elisa kits and (**c**) RECK expression levels, by Western Blots, were determined in kidney cortex of rats submitted to partial liver ischemia (60 min) followed by reperfusion (120 min). Sham-operated animals were subjected to the same procedure without clamping the vessels. *n* = 6 rats/group. Results are expressed as median value (red line) ± error bar (1–99 percentiles) (black dot). one-way ANOVA with Tukey–Kramer test (MMP-9-dimer, MMP-2, and TIMP-1); Kruskal–Wallis test with Conover test (TIMP-2 and RECK). * *p* < 0.05 versus I/R rats.

The same trend in MMP-9-dimer activity occurred in the medulla obtained from the I/R group and I/R group treated with OCA (Figure 2a).

Although not significantly, the MMP-9-dimer protein trend was superimposable with MMP-9-dimer activity. In the cortex, MMP-9-dimer protein increased in I/R by 1.3-fold and in OCA-treated I/R by 0.8-fold. In the medulla, MMP-9-dimer protein increased in I/R by 1.8-fold and in OCA-treated I/R by 1.2-fold. Our data are expressed as a fold increase in their respective sham controls. Tubulin levels, which served as a loading control, remained unchanged (Figure 1a).

Although not significantly, cortex MMP-2 activity also increased after hepatic I/R compared with sham-operated rats and decreased in OCA-treated I/R rats (Figure 1a).

No changes were found in medulla MMP-2 activity in any group (Figure 2a).

In the cortex, MMP-2 protein increased in I/R by 1.2-fold and in OCA-treated I/R by 1.2-fold. In the medulla, MMP-2 protein increased in I/R by 0.8-fold and in OCA-treated I/R by 0.8-fold. Our data are expressed as a fold increase in their respective sham controls. MMP-2 protein evaluation documented no difference comparing I/R versus I/R + OCA both in the cortex and medulla. Tubulin levels, which served as a loading control, remained unchanged (Figure 2a).

Matrix metalloproteinases are inhibited by specific TIMPs; in addiction, RECK represents a novel matrix metalloproteinase regulator. The evaluation of TIMP-1 and TIMP-2 expression levels showed no significant differences among all groups considered (Figure 1b and Figure 2b) and the same trend occurred for RECK expression levels (Figure 1c and Figure 2c) both in the cortex and medulla.

### 2.3. OCA Treatment Reduces Serum Levels of Creatinine

**Figure 3:**
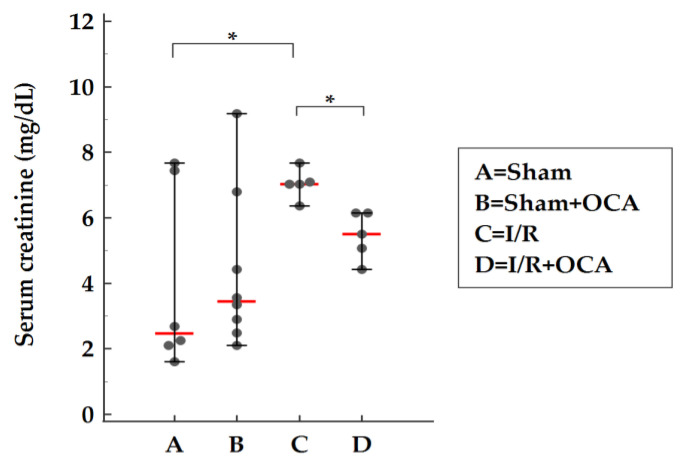
OCA treatment decreases serum creatinine in rats submitted to partial liver ischemia (60 min) followed by reperfusion (120 min). Animals were orally administered 10 mg/kg/day of OCA in methylcellulose 1% vehicle for 5 days (*n* = 12) or vehicle alone (*n* = 12). Sham-operated animals were subjected to the same procedure without clamping the vessels. *n* = 6 rats/group. Results are expressed as median value (red line) ± error bar (1–99 percentiles) (black dot). one-way ANOVA with Tukey–Kramer test. * *p* < 0.05 versus I/R rats.

The serum creatinine concentration is widely interpreted as a measure of the glomerular filtration rate (GFR) and is used as an index of renal function in clinical practice [14]. Serum creatinine levels were evaluated in rats submitted to liver I/R: an increase was found after I/R when compared with sham animals and a significant reduction in sham-operated levels was detected in OCA-treated I/R rats (Figure 3).

### 2.4. OCA Treatment Reduces Kidney Cortex Levels of TNF-Alpha and IL-6

**Figure 4:**
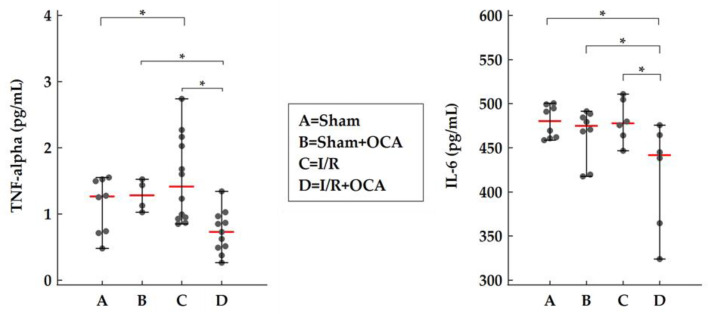
OCA treatment decreases cortex TNF-alpha and IL-6 in rats submitted to partial liver ischemia (60 min) followed by reperfusion (120 min). Animals were orally administered with OCA 10 mg/kg/day in methylcellulose 1% for 5 days (*n* = 12) or with vehicle alone (*n* = 12). Sham-operated animals were subjected to the same procedure without clamping the vessels. *n* = 6 rats/group. Results are expressed as median value (red line) ± error bar (1–99 percentiles) (black dot). Kruskal–Wallis test with Conover test. * *p* < 0.05 versus I/R rats.

The effect of OCA on kidney TNF-alpha and IL-6 levels was evaluated by ELISA. At the end of reperfusion, OCA administration caused a significant decrease in both TNF-alpha and IL-6 in the kidney cortex of OCA-treated I/R rats compared to I/R rats (Figure 4). Undetectable levels of TNF-alpha were found in the kidney medulla in all groups considered. No changes in the IL-6 kidney medulla were found (Figure S1, supplementary materials).

### 2.5. FXR Expression in the Kidney

**Figure 5:**
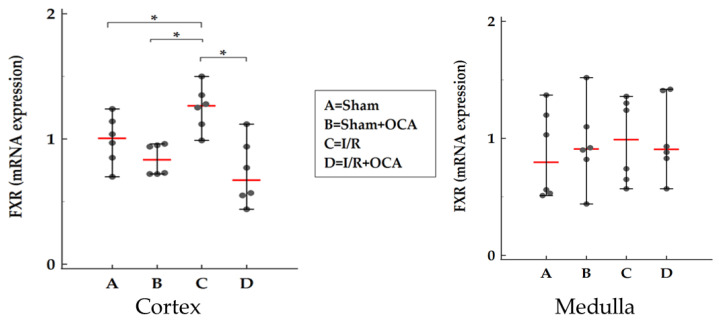
FXR expression in kidney cortex and medulla in rats submitted to partial liver ischemia (60 min) followed by reperfusion (120 min). Animals were orally administered with OCA 10 mg/kg/day in methylcellulose 1% for 5 days (*n* = 12) or with vehicle alone (*n* = 12). Sham-operated animals were subjected to the same procedure without clamping the vessels. *n* = 6 rats/group. Results are expressed as median value (red line) ± error bar (1–99 percentiles) (black dot). One-way ANOVA with Tukey–Kramer test; * *p* < 0.05 versus I/R rats.

The kidney itself expresses FXR both in the cortex and in the medulla as shown in Figure 5. After OCA treatment a decrease in FXR expression occurred only in the cortex.

### 2.6. TBARS Formation in the Kidney

**Figure 6:**
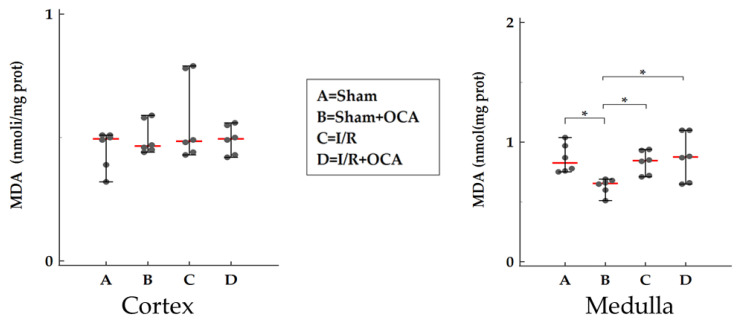
TBARS formation in kidney cortex and medulla in rats submitted to partial liver ischemia (60 min) followed by reperfusion (120 min). Animals were orally administered with OCA 10 mg/kg/day in methylcellulose 1% for 5 days (*n* = 12) or with vehicle alone (*n* = 12). Sham-operated animals were subjected to the same procedure without clamping the vessels. *n* = 6 rats/group. TBARS concentrations were calculated using malondialdehyde (MDA) as standard. Results are expressed as median value (red line) ± error bar (1–99 percentiles) (black dot). One-way ANOVA with Tukey–Kramer test (Medulla); Kruskal–Wallis test with Conover test (Cortex). * *p* < 0.05 versus I/R rats.

We evaluated the kidney concentration of TBARS after 2-h reperfusion both in the cortex and in the medulla (Figure 6). No significant changes in lipid peroxidation were detected for all groups considered in the cortex (Figure 6) and no difference in I/R groups in the medulla (Figure 6).

### 2.7. OCA Treatment and Histological Changes in the Kidney

**Figure 7:**
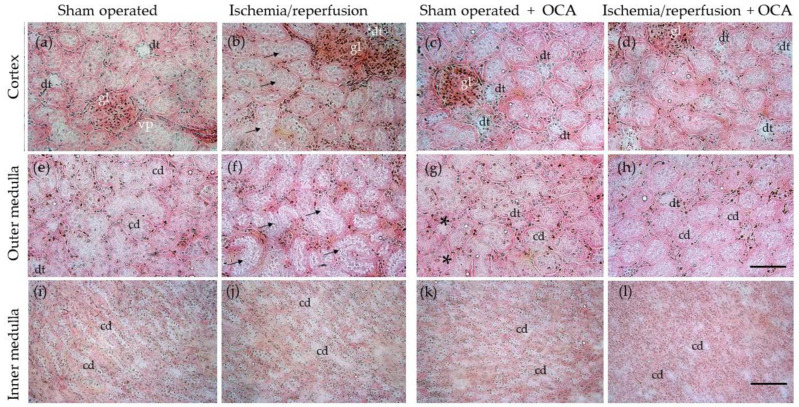
Histological patterns of kidneys (cortex and outer and inner medulla) collected from rats orally administered 10 mg/kg/day OCA in methylcellulose 1% for 5 days (*n* = 12) or vehicle alone (*n* = 12), and then submitted to partial liver ischemia (60 min) followed by reperfusion (120 min). Sham-operated animals were subjected to the same procedure without clamping the vessels. Cryostatic tissue sections are stained by conventional H&E procedure. (**a**–**d**) In the cortex the intensely stained glomeruli, or renal corpuscles, (gl) exhibit their typical rounded shape. In (**a**) the vascular pole (vp) is also easily appreciable. Distal tubules (dt) can be recognized for their much less eosinophilia than the proximal ones. (**e**–**h**) Cross-sections of medulla are characterized by collecting ducts (cd), larger than distal tubules (dt) and segments of the loop of Henle (asteriscs). (**i**–**l**) In the longitudinal sections of medulla, arrays of nuclei facing larger, empty spaces identify collecting duts (cd). Ischemia-reperfusion affects in particular the tubules in the cortex and outer medulla of rats without OCA administration, for which the lumen appears to be enlarged and filled with amorphous material (arrows). Bars: (**a**–**h**) 80 μm; (**i**–**l**) 50 μm.

Examples of histological patterns of kidneys collected from vehicle-treated I/R and OCA-treated I/R rats after 60 min of liver ischemia and 120 min reperfusion are shown in Figure 7. In rats submitted to liver I/R, the kidney shows dilated tubules, in particular in the cortex and outer medulla (Figure 7b,f), compared to the respective sham-operated controls (Figure 7a,e). This morphological alteration is not appreciable in kidneys from OCA-treated rats submitted to I/R (Figure 7d,h).

Collagen I was evaluated by Western blot: collagen I was not detectable in all groups considered (Figure S2, supplementary materials).

## 3. Discussion

### 3.1. Hepatic Ischemia/Reperfusion and Renal Damage

Acute I/R-induced liver failure is a known clinical problem and often leads to remote organ dysfunction including lung, heart, and kidney [17]. Particularly, acute kidney injury (AKI), associated with liver failure, leads to hepatorenal syndrome, a serious clinical problem characterized by a high mortality rate. The pathogenesis of AKI associated with liver failure is still poorly understood, also due to the lack of reproducible and reliable animal models. In this study, we used a rat acute I/R-induced liver failure model to mimic biochemical (serum creatinine, MMP, TIMP, inflammatory markers such as TNF-alpha and IL-6, lipid peroxidation, and fibrosis) and histological (renal tubular damage as indicated by dilatation) changes observed in a hepatorenal syndrome. Through the reduction of renal matrix metalloproteinases, in particular gelatinases, our study suggests that OCA treatment can ameliorate hepatic renal syndrome following partial hepatic I/R injury in rats.

In the last decade, the Acute Kidney Injury Network (AKIN) has proposed new diagnostic criteria for AKI, based on small changes in serum creatinine levels [18]. Traditionally, kidney failure in cirrhosis has been defined as serum creatinine above 1.5 mg/dL [12]. The definition of AKI in cirrhosis has recently been changed and is based on small changes in serum creatinine associated with urinary biomarkers and pro-inflammatory cytokines [19]. In our data hepatic ischemia increased serum creatinine levels by 1.8-fold to 7.35 mg/dL, indicating acute renal failure, and thus validating our model of AKI mediated by liver I/R injury.

Molecular mechanisms underlying I/R injury involved reactive oxygen species (ROS) formation released by Kupffer cells, adherent leukocytes, or mitochondrial sources. Liver I/R injury may initiate a systemic inflammatory response that promotes remote organ dysfunctions attributed to oxidative stress mediators and other remotely released factors, including proinflammatory cytokines, tumor necrosis factor, and interleukins [8]. Oxidative stress is considered a major determinant of liver I/R induced AKI. Activated neutrophils, ROS, and cytokines release cause direct renal injury and the recruitment of monocytes and macrophages leading to further aggravation of the oxidative injury [8]. The pivotal role of ROS in the development of AKI is also demonstrated by the positive effect of the administration of free radical scavengers such as quercetin and desferrioxamine that protected the kidney by decreasing TBARS levels [20]. We evaluated the kidney concentration of TBARS after 2-h reperfusion and no changes in lipid peroxidation were detected, thus suggesting that in our model, ROS generation is not underlying the early remote damage in the kidney.

### 3.2. Renal Damage Is Associated to MMPs Activation

Most matrix metalloproteinases (MMPs), zinc-containing endopeptidases involved in the extracellular matrix (ECM) remodeling [21], and their specific tissue inhibitors (TIMPs) are expressed in the kidney even if their spatial expression is complex and has not been completely characterized [22]. MMPs are associated with both physiological and pathological processes in the kidney [23]. Knockout mouse models have provided insights into the cause-and-effect relationship between MMP activity and renal pathophysiology [24,25,26]. There is now convincing evidence that MMPs could have both pathogenic and nephroprotective effects in acute and chronic kidney diseases.

We have previously documented that moderate acute hepatic ischemia (30 min) followed by reperfusion (60 min) increases MMPs activity not only in the ischemic liver region but also in the lung, associated with histological damage in the liver, lung, and kidney [9]. Additionally, no significant difference in MMPs was observed in other distant organs such as the kidney and heart. The short duration of both the ischemic (30 min) and reperfusion periods (60 min) of our experimental model could represent a possible explanation for these results. Thus, we designed a new study to evaluate whether liver I/R may induce renal MMPs activation in rats. A liver ischemia (60 min) followed by a longer reperfusion (120 min) was associated with a significant MMP-9-dimer activity increase in the kidney cortex and medulla. The result obtained show how even the increased activity of MMP-9 dimer can be inserted in a context of renal damage characterized by a significant increase in creatinine and a histological picture of mild damage.

Zymography analysis revealed the presence of gelatinolytic activities of MMP-9 dimer, detected at about 220 kDa, and MMP-2 at 68 KDa. No MMP-9 monomers were detected in kidney tissue. MMP-9, in contrast to MMP-2, exists in two major forms: a monomeric (92 kDa) and a disulfide-bonded homodimeric (220 kDa) form. MMP-9 dimer has been identified in a variety of MMP-9-producing cells including neutrophils and normal breast epithelial cells [27]. Enzymatic activity of the monomeric and dimeric forms of MMP-9 have different biochemical and enzymatic properties. The monomer is more rapidly activated by MMP-3 and has a higher activity than the dimer [28]. The existence of the more stable, slow-activating MMP-9 dimer might serve as a regulatory mechanism during ECM degradation [28]. In the present study, although not significantly, kidney MMP-2 activity increases. Caron et al. reported similar results in glomerular tissue in a rat model of renal ischemia-reperfusion. The most marked effect revealed by this study was a higher MMP-9 dimer induction. Authors reported that protein expression of dimeric MMP-9 forms was highly stimulated (about 8-fold) by ischemia, compared to the expression of MMP-2 (1.5-fold). It may be possible that a reduced MMP-2 expression may be accompanied by less detectable activity.

Several studies [29,30,31,32] have shown protective effects against AKI with MMP inhibitors, but there are data from knockout mouse studies where MMP-2 and -9 may show both proinjury and nephroprotective effects [24].

Loss of MMP-2 is not equivocally protective, while MMP-9 deficiency may exacerbate an injury. There is also evidence that MMP-2 may be important for recovery following injury, while MMP-9 overexpression may cause disrepair through microvascular loss. It is known that MMP-9 is an acknowledged early marker of acute renal damage [16]. These data indicate that more research remains to be performed to elucidate these pathogenic mechanisms.

### 3.3. OCA Treatment Reduces Kidney Gelatinases Activity following Partial Hepatic I/R Injury in Rats

In the present study, in order to characterize the mechanisms of remote organ damage, we investigated the effects of OCA on kidney levels of gelatinase, MMP-2, and MMP-9.

Very few studies have addressed the ability of OCA to modulate the activity of metalloproteases [6,33]. Recently, our group has detected the ability of OCA to limit the activation of MMP-2 and MMP-9 occurring during hepatic I/R damage, probably via a TIMP- and RECK-mediated mechanism [6].

Liver I/R injury is associated with a systemic inflammatory response that promotes remote organ dysfunctions. In the present study, a reduction in MMP-9-dimer was detected both in the kidney cortex and in the medulla of OCA-treated I/R rats. In this animal model of remote organ injury, we did not find a corresponding modulation of MMP and RECK inhibitors as in the I/R liver. It can be assumed that the modulation of OCA gelatinolytic activity is likely to be mediated by tissue TNF-alpha and IL-6. Indeed, treatment with OCA at 120 min caused a significant decrease in both TNF-alpha and IL-6 in the kidney cortex. A wealth of evidence indicates that circulatory dysfunction plays a key role in the pathophysiology of hepatorenal syndrome [34]. However, recently, evidence has emerged to support the impact of systemic inflammation on disease progression and development in extrahepatic organs, particularly renal dysfunction [35]. After liver I/R injury, circulating pro-inflammatory cytokines such as interleukin-6 (IL-6), IL-8, TNF-alpha, vascular cell adhesion protein 1 (VCAM-1), fractalkine, and macrophage inflammatory protein-1 alpha (MIP-1 alpha) have been found significantly increased [36].

Pro-inflammatory cytokines such as TNF-alpha and IL-6 are produced in response to infection. During hepatic I/R, serum TNF-alpha has been shown to act as one of the key mechanisms in modulating MMP activity in remote organs [9]. Tissue TNF-alpha may be a possible additional source for TNF-alpha traced in the serum in our previous work using the same animal model. In this study, we detected TNF-alpha in cortical renal tissue and found an increase in this proinflammatory cytokine in rats undergoing I/R, readily modulated by OCA administration. The decrease in cortex IL-6 by OCA administration supports its role in the modulation of inflammatory response.

However, sham animals reveal high levels of tissue IL-6 and TNF-alpha probably due to the operative trauma of laparotomy as already reported by Ogura et al. [37] and Wanner G et al. [38].

It is known that an excessive cytokine release enhances vascular permeability and impairs metabolic processes, thus increasing susceptibility to multiple organ dysfunction [36,39].

The evaluation of TIMP levels showed no significant differences among all groups considered and the same occurred for RECK. In our research, we also determined the content of both TIMP-1, the specific inhibitor of MMP-9, and TIMP-2, the inhibitor of MMP-2. Both inhibitors showed no significant differences among all groups tested. Usually, TIMP concentration is opposite to MMP. However, in our study, we found increased concentrations of both MMP-9 and TIMP-1, although not significant for the latter; this could result from failure of TIMP-1 inhibition. Furthermore, TIMP transcription is regulated by the same cytokines and growth factors controlling MMP expression [40]. The TIMP-2 concentration in the I/R group did not have a statistically significant influence but it showed, as expected, a negative trend in contrast with MMP2.

MMPs are also inversely regulated by RECK, a membrane-anchored glycoprotein and a key regulator of ECM integrity [6,41]. In this study, the ability of OCA to restore renal TIMP and RECK expression was lacking. Although not statistically significant, in the kidney cortex it was still possible to detect a modest increase in both TIMP-1 and TIMP-2 in OCA I/R treated group. Probably, the ability of OCA to reduce kidney matrix metalloproteinase activation following hepatic I/R injury in rats is not modulated by RECK and TIMP. Thus, we suppose that OCA protection is principally due to the reduction of the inflammatory response.

The kidney itself expresses FXR in the cortex and the medulla. No significant changes in FXR levels occurred in the sham and I/R group after 2-h reperfusion in agreement with Ogura et al.; they documented no changes in FXR expression remote damage occurred in the liver after intestinal ischemia followed 1 and 3-h reperfusion [37]. After OCA treatment a decrease in FXR expression occurred only in the cortex; this event will be further investigated in future studies with the reperfusion time prolonged.

The present study also demonstrated that OCA treatment alleviates I/R-induced kidney damage as found by a marked decrease in serum creatinine levels. Furthermore, the histological analysis in the cortex and outer medulla in rats submitted to I/R, reveals some alterations, such as dilated tubules, as compared with the sham-operated animals. These morphological changes are less noticeable in kidneys from OCA administered rats submitted to liver I/R.

Our results show that, after hepatic I/R injury, hepatorenal syndrome rapidly develops in rats, characterized by an increase in MMP-9-dimer and inflammatory changes such as an increase in renal cortex TNF-alpha, thus suggesting a key role for this pro-inflammatory cytokine in MMP activation. Although the underlying mechanism needs further investigation, this study shows, in the kidney, beneficial effects of OCA by reducing TNF-alpha-mediated expression of MMPs after liver I/R.

## 4. Materials

All reagents were of the highest grade of purity available and were obtained from MERCK—Life Science, 20149 Milan, Italy). The FXR agonist, OCA, was kindly provided by Intercept Pharmaceuticals, San Diego, CA, USA.

### 4.1. Animals and Experimental Design

The use and care of animals in this experimental study was approved by the Italian Ministry of Health and by the University of Pavia Commission for Animal Care (Document number 179/2017-PR). Male Wistar rats (200–250 g) (Charles River, 23885 Calco, LC, Italy) were used in this study. The animals (*n* = 24) were allowed free access to water and food in all the experiments. Animals were orally administered with 10 mg/kg/day OCA in methylcellulose 1% for 5 days (*n* = 12) or with vehicle alone (*n* = 12). The effects of I/R-induced remote damage in kidneys were studied in vivo using a partial hepatic I/R model (I/R *n* = 12 and I/R + OCA *n* = 12). The rats were anesthetized with sodium pentobarbital (40 mg/kg i.p.), the abdomen was opened via a midline incision and the bile duct was cannulated (PE-50) [17]. Ischemia to the left and the median lobe was induced for 60 min with microvascular clips by clamping the branch of portal vein and the branch of the hepatic artery after the bifurcation to the right lobe, with the abdomen temporarily closed with a suture [18]. After a 60-min ischemia, the abdomen was reopened, the clips were removed, the abdomen was closed again, and the liver was allowed to reperfuse for 120 min (Figure 1). By using partial, rather than total, hepatic ischemia, portal vein congestion, and subsequent bacterial translocation into the portal venous blood were avoided. Sham-operated animals were subjected to the same procedure without clamping the vessels (sham-operated *n* = 6 and sham-operated + OCA *n* = 6). To prevent postsurgical dehydration and hypotension, 1 mL of saline was injected into the inferior vena cava. All the animals were maintained on a warm support to prevent heat loss, rectal temperature was maintained at 37 ± 0.1 °C. Animals were sacrificed, under general anesthesia (sodium pentobarbital 40 mg/kg i.p.), by exsanguination. Each sample is derived from individual kidneys.

Blood samples were obtained after reperfusion and immediately centrifuged to isolate serum. At the end of the reperfusion period, tissue samples of the kidney (cortex and medulla) were snap-frozen in liquid nitrogen.

### 4.2. Biochemical Assays

Liver injury was assessed by transaminase serum release (alanine transaminase, ALT, and aspartate transaminase, AST); alkaline phosphatase (ALP), total and direct bilirubin were assayed using commercial kits, MERCK—Life Science, 20149 Milan, Italy.

Kidney injury was assessed by serum creatinine colorimetric assay kit (number 700460, Cayman Chemicals, Ann Arbor, MI 48108, USA). The kit was performed following manufacturers’ instructions.

### 4.3. TBARS Formation

The extent of lipid peroxidation was evaluated, as previously described [42], by measuring the formation of thiobarbituric acid-reactive substances (TBARS), following the Esterbauer and Cheeseman [43] method. TBARS concentrations were calculated using malondialdehyde (MDA) as standard.

### 4.4. Tissue Sources for MMPs Analysis

After sacrifice, kidneys were quickly excised and placed in cold (4 °C) buffer (30 mM Histidine, 250 mM sucrose, and 2 mM EDTA, pH 7.2) to remove blood. The kidney was cleaned of external tissue; the renal cortex and medulla were separated and subsequently frozen in liquid nitrogen and stored at −80 °C, until use. Fifty milligrams of cortex and medulla were homogenized in a dissociation buffer containing 10 mmol/L cacodylic acid, 0.15 mmol/L NaCl, 1 mmol/L ZnCl_2_, 20 mmol/L CaCl_2_, 1.5 mmol/L NaN_3_, and 0.01% Triton X-100, pH 5.0 [25]. The homogenate was then shaken at 4 °C for 24 h and the protein concentration of the supernatant was measured with the colorimetric Lowry method [24]. Samples were stored at −20 °C before use.

### 4.5. MMP Zymography

In order to detect MMPs activity present in the samples, the homogenate protein content was normalized by a final concentration of 400 μg/mL in sample loading buffer (0.25 M Tris-HCl, 4% sucrose *w*/*v*, 10% SDS *w*/*v*, and 0.1% bromophenol blue *w*/*v*, pH 6.8). After dilution, the samples and purified MMP-9 dimer, MMP-9, and MMP-2 (Enzo Lifescience and Calbiochem-Merck Life Science, 20149 Milan, Italy) were loaded onto electrophoretic gels (SDS-PAGE) containing 1 mg/mL of gelatin under nonreducing conditions [27,28], followed by zymography as described previously [29]. Zymogram experiment was replicated 3 times for each sample.

The zymograms were analyzed by densitometer (GS 710 Densitometer BIORAD, Hercules, CA, USA) and data were expressed as optical density (OD), reported to 1 mg/mL protein content.

### 4.6. Western Blots

CelLytic Buffer and Protease Inhibitor Cocktail were purchased from Sigma-Aldrich (Milan, Italy), as well as the monoclonal antibody anti-alpha-tubulin (DM1A). Rabbit monoclonal antibody against RECK (D8C7) was purchased from Cell Signaling Technology (Euroclone, Milan, Italy). Rabbit polyclonal antibody against Collagen I alpha 1 (NBP1-30054) was purchased from Novus Biological (Bio-techne, Milan, Italy). Mouse monoclonal antibody against MMP-2 and MMP-9 were purchased by Thermo Fisher Scientific (Monza, Italy).

Kidney tissue samples were homogenized in an ice-cold CelLytic Buffer supplemented with Protease Inhibitor Cocktail and centrifuged at 15,000× *g* for 10 min. The collected supernatant was divided into aliquots containing the same amount of proteins and stocked at −80 °C. Samples of kidney extracts containing the same amount of proteins were diluted with 2× Laemmli sample buffer purchased by BIO-RAD (Segrate, Italy) added with 0.1 M DTT (Sigma-Aldrich, Milan, Italy). Samples were incubated for 10 min at 70 °C and then put for 1 min on ice to cause thermal shock. Samples used for the evaluation of MMP-9 dimer were loaded under unreducing conditions: they were diluted with 2× Laemmli sample buffer without reducing agent, and were not heated at 70 °C. No thermal shock was caused to them. Thus, samples of kidney extracts containing the same amount of proteins were separated in SDS-PAGE on 7.5% acrylamide gels and transferred to PVDF membrane. Unspecific sites were blocked for 2 h with 5% Bovine Serum Albumin (BSA) in TBST (20 mM Tris/Base, 150 mM NaCl, 7.4 pH, 0.1% Tween 20) at 4 °C. The membranes were incubated with primary antibodies overnight at 4 °C, under gentle agitation. Primary antibodies against alpha-tubulin and RECK were used at a dilution of 1:1000. Membranes were washed in TBST (20 mM Tris/Base, 150 mM NaCl, pH 7.4, 0.1% Tween 20) and incubated with peroxidase-conjugated secondary antibody at a 1:2000 dilution for both tubulin and RECK. Primary antibodies against alpha-tubulin and Collagen I Alpha 1 antibody were used at a dilution of 1:1000 and membranes were washed in TBST (20 mM Tris/Base, 150 mM NaCl, pH 7.4, 0.1% Tween 20) and incubated with peroxidase-conjugated secondary antibody at a 1:5000 diluition for both tubulin and Collagen I Alpha 1. Immunostaining was revealed with BIO-RAD Chemidoc XRS+. Bands intensity quantification was performed by BIO-RAD Image Lab software, 20090 Segrate, MI, Italy.

### 4.7. TIMP-1, TIMP-2, TNF-Alpha, and IL-6 Enzyme-Linked Immunosorbent Assay

Kidney Cortex and Medulla were homogenized with suitable lysis buffer as indicated by the collection procedures of the kit suppliers. After centrifuging the tissue homogenates for 5 min at 10.000 rpm, supernatants were collected and the concentration of TIMP-1 (Abnova), TIMP-2 (Abnova), and TNF-alpha and IL-6 (Antibodies-online GmbH, Aachen, Germany) was immediately measured by ELISA kit.

### 4.8. FXR Expression

FXR mRNA expression was analyzed by a real-time polymerase chain reaction (RT-PCR). Total RNA was isolated from both kidney cortex and medulla homogenized in TRI reagent (Sigma-Aldrich, St. Louis, MO, USA), in accordance with the method described by Chomczynski et al. (1995) [44]. RNA quantification was evaluated by measuring the absorbance at 260 nm with a T92^+^ UV Spectrophotometer (PG Instruments, Lutterworth, UK); the RNA purity was also evaluated by calculating the 260/280 nm ratio. iScript Supermix (Bio-Rad, Milan, Italy) was employed to generate the cDNA [45]. mRNA expression was analyzed using the SsoAdvancedTM SYBR^®^ Green Supermix (Bio-Rad, Milan, Italy) and the amplification was performed through two-step cycling (60–95 °C) for 40 cycles in a CFX96TMReal-TimeSystem (Bio-Rad, Milan, Italy). FXR, ubiquitin C (UBQ), and glyceraldehyde 3-phosphate dehydrogenase (GAPDH) gene amplification efficiency (99.8%, 100%, 102.8%, respectively), was established by means of calibration curves, in a cDNA concentration range of 10–0.625 ng/μL. As regards housekeeping genes, UBC and GAPDH were used. The sequence of forward and reverse primers used in the experiments is reported in Table 2. All samples were assayed in duplicate and the expression of the reference genes remained constant in all the experimental groups. Gene expression was calculated using the ΔCt method. Comparison between groups was calculated using the ΔΔCt method.

### 4.9. Tissue Morphology

Kidney tissue samples collected after animal sacrifice were frozen in liquid nitrogen and stored at −80 °C until they were cut at cryostat (CM1850, Leica Microsystems, GmbH, Wetlzar, Germany). Tissue sections (12 μm thick) were air-dried for at least 24 h and stained following conventional hematoxylin eosin (H&E) procedure. Tissue morphology was observed using an Olympus BX51 microscope (Olympus Optical Co. GmBH, Hamburg, Germany) equipped with a Canon EOS 1300D (Canon Inc. Ōta, Tokyo, Japan) digital photo camera, and an Olympus 20× UplanFl objective (NA 0.50 Olympus Optical Co. GmbH, Hamburg, Germany).

### 4.10. Statistical Analysis

Statistical analysis was performed using MedCalc Statistical Software version 18.11.3 (MedCalc Software bvba, Ostend, Belgium; https://www.medcalc.org; accessed on 1 March 2022). Statistical analysis was performed with one-way ANOVA with Tukey’s test, as post hoc test, or Kruskall–Wallis with Conover’s test when data were not normally distributed. To assess normality of distribution, Kolmogorov–Shapiro normality test was used. Results are expressed as mean value ± standard error (SE) or median value with range percentiles. The value of *p* = 0.05 was considered the criterion for statistical significance.

## 5. Conclusions

This is the first study reporting that the pretreatment with OCA resulted in attenuation of renal injury after liver I/R by reducing TNF-alpha-and IL6 mediated expression of MMPs.

## Figures and Tables

**Table 1:** Serum biochemical parameters in sham and I/R rats and creatinine/ALT relationship.

	Sham	I/R
AST (mU/mL)	256 ± 33	9653 ± 956 *
ALT (mU/mL)	64 ± 10	8635 ± 847 *
ALP (mU/mL)	459 ± 50	803 ± 55 *
Total Bilirubin (mg/dL)	0.15 ± 0.01	0.31 ± 0.02 *
Direct Bilitubin (mg/dL)	0.05 ± 0.01	0.21 ± 0.01 *
Creatinine (mg/dL)	3.96 ± 1.10	7.35 ± 0.40 *
	r	*p*
Creatinine/ALT	0.94	0.002

*n* = 6 rats/group. Results are expressed as mean value ± standard error (SE). one-way ANOVA with Tukey–Kramer test. * *p* < 0.05 versus sham rats.

**Table 2:** List of forward and reverse primers used in the experiments.

Gene	Sequence
*rat FXR*	Forward: CGCCTCATCGGCGGGAAGAA
	Reverse: TCACGCAGTTGCCCCCGTTC
*rat GAPDH*	Forward: AACCTGCCAAGTATGATGAC
	Reverse: GGAGTTGCTGTTGAAGTCA
*rat UBQ*	Forward: CACCAAGAAGGTCAAACAGGAA
	Reverse: AAGACACCTCCCCATCAAACC

## Data Availability

Data is contained within the article and its supporting information.

## Supplementary Materials

The following supporting information can be downloaded at: https://www.mdpi.com/article/10.3390/ph15050524/s1. Figure S1. IL-6 in rats submitted to partial liver ischemia (60 min) followed by reperfusion (120 min). Animals were orally administered with OCA 10 mg/kg/day in methylcellulose 1% for 5 days (*n* = 12) or with vehicle alone (*n* = 12). Sham-operated animals were subjected to the same procedure without clamping the vessels. *n* = 6 rats/group. Results are expressed as median value ± error bar (1–99 percentiles). Kruskal-Wallis test with Conover test. Figure S2. Collagen I expression levels, by Western Blots, were determined in kidney cortex (a) and medulla (b) of rats submitted to partial liver ische-mia (60 min) followed by reperfusion (120 min). Sham-operated animals were subjected to the same procedure with-out clamping the vessels. *n* = 6 rats/group.
