# Supplementary material for: Obeticholic Acid Reduces Kidney Matrix Metalloproteinase Activation Following Partial Hepatic Ischemia/Reperfusion Injury in Rats
Source: Pharmaceuticals (Basel). 2022 Apr 24;15(5):524. doi: 10.3390/ph15050524 (PMC9145209; doi:10.3390/ph15050524)
Supplement: Supplementary file 1 [file pharmaceuticals-15-00524-s001.zip › pharmaceuticals-1691605-supplementary.pdf]

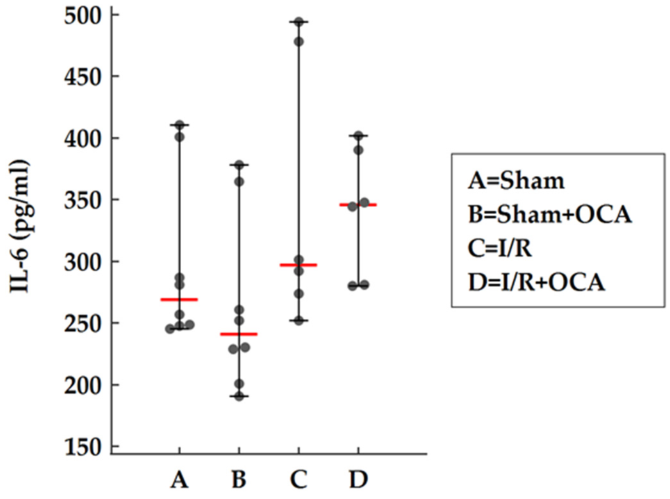

Figure S1. IL-6 in rats submitted to partial liver ischemia (60 min) followed by reperfusion (120 min). Animals were orally administered with OCA 10 mg/kg/day in methylcellulose 1% for 5 days (n=12) or with vehicle alone (n=12). Sham-operated animals were subjected to the same procedure without clamping the vessels. n=6 rats/group. Results are expressed as median value ± error bar (1-99 percentiles). Kruskal-Wallis test with Conover test.

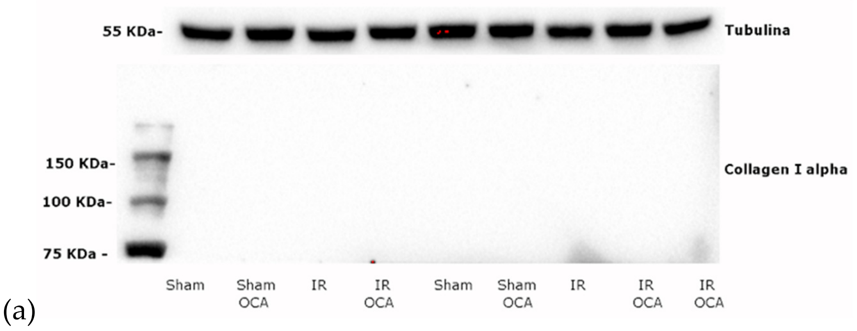

(a)

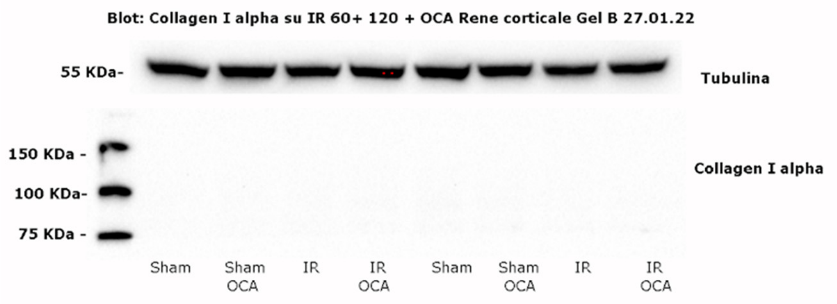

(b)

Figure S2. Collagen I expression levels, by Western Blots, were determined in kidney cortex (a) and medulla (b) of rats submitted to partial liver ische-mia (60 min) followed by reperfusion (120 min). Sham-operated animals were subjected to the same procedure with-out clamping the vessels. n=6 rats/group.
